# Cross-national comparison of gender differences in the enrollment in and completion of science, technology, engineering, and mathematics Massive Open Online Courses

**DOI:** 10.1371/journal.pone.0202463

**Published:** 2018-09-13

**Authors:** Suhang Jiang, Katerina Schenke, Jacquelynne Sue Eccles, Di Xu, Mark Warschauer

**Affiliations:** 1 School of Education, University of California Irvine, Irvine, California, United States of America; 2 Graduate School of Education & Information Studies, University of California Los Angeles, Los Angeles, California, United States of America; Universidad Nacional de Educacion a Distancia (UNED), SPAIN

## Abstract

Massive Open Online Courses (MOOCs) have the potential to democratize education by providing learners with access to high-quality free online courses. However, evidence supporting this democratization across countries is limited. We explored the question of MOOC democratization by conducting cross-national comparisons of gender differences in the enrollment in and completion of science, technology, engineering, and mathematics (STEM) MOOCs. We found that while females were less likely than males to enroll in STEM MOOCs, they were equally likely to complete them. Further, a higher probability to enroll in STEM MOOCs and smaller gender gaps in STEM MOOC enrollment and completion were found in less gender-equal and less economically developed countries.

## Introduction

Massive Open Online Courses (MOOCs) have attracted tens of millions of learners around the world. Theoretically, anyone with an Internet connection is able to freely access these online courses, which are often provided by professors from elite universities. Similar to previous technological advancements in broadcast media, such as radio and television, MOOCs were expected to transform education by providing learning opportunities for those who otherwise would not have access to them [1]. The growing MOOC movement stems from the beliefs that knowledge should be freely shared and people have the right to learn regardless of their social and economic backgrounds [1]. MOOC proponents argue that MOOCs can democratize higher education and provide learning opportunities not only for traditionally underserved populations but also for college-educated populations, since both may improve their employment opportunities through the extra coursework provided [2].

However, the optimistic expectation that MOOCs will promote educational equity has been dampened by studies describing the demographics of individuals who enroll in and complete MOOCs [3–5]. Statistics show that the majority of MOOC learners are young, well-educated males from developed countries [3]. In the United States, for example, individuals of higher socioeconomic status (SES) are much more likely to enroll in MOOCs than people of lower SES [5]. Based on these demographics, critics argue that MOOCs are failing to reach disadvantaged individuals, such as those without access to higher education in developing countries [6]. This critique implicitly assumes that those in developing countries who have already earned a college degree should not be considered disadvantaged. However, compared to their peers from developed countries, those in developing countries who already have a college degree are still at a disadvantage in terms of both accessing high-quality education from elite universities and high-quality jobs that often result from such an elite education.

In addition to the critique that MOOCs do not reach disadvantaged individuals, concerns have been voiced about whether MOOCs increase the participation of females in STEM fields [4]. Gender disparity is prevalent in MOOCs, especially in STEM subjects. On average, only 1 in 5 learners in a STEM MOOC is female [4]. As females have been traditionally underrepresented in STEM fields, we are particularly interested in females’ enrollment and performance in STEM MOOCs. For example, females constitute 29% of those employed in science and engineering occupations in the United States [7], 12.8% in the United Kingdom [8], 16% in Australia [9], and 13.8% in Japan [10]. Increasing female participation in STEM fields is crucial for strengthening the STEM workforce and for a country’s global competitiveness [7]. Though females are generally underrepresented in STEM MOOC participation, it is unclear whether the gender disparity differs across countries and, if so, how. No studies have explored how country-level characteristics (e.g., gender equality and economic development level) may moderate the relationship between gender and the enrollment in and completion of STEM MOOCs. Investigating the moderating effect of country-level characteristics would provide evidence either for or against the claim that MOOCs are democratizing higher education across the world.

Therefore, this paper aims to explore the question of MOOC global democratization by examining the cross-national differences of females’ enrollment in and completion of STEM MOOCs and exploring whether and how the size of the gender gap in STEM MOOC enrollment and completion varies by country-level characteristics (e.g., gender equality and economic development level). We specifically examine enrollment and completion separately because MOOCs are notorious for having very low completion rates [4]. Additionally, different factors may be associated with whether an individual decides to enroll in a STEM MOOC and whether that individual actually completes it.

### Related work

Our analytical framework is guided by the Eccles’ Expectancy-Value Model of Achievement-Related Choices [11–13]. This model accounts for individuals’ choices of and performance in activities [13]. It suggests that social context and cultural forces contribute to gendered educational choices [12,13]. Gender role stereotypes and cultural stereotypes of subject matter and occupational characteristics influence individuals’ achievement choices through the socialization process [14]. In addition, we consider the impact of economic development level on females’ educational choices because economic development has been found to be associated with less gender segregation and more gender equality in education [15,16]. We reviewed the literature on cross-national differences in STEM education enrollment and performance, gender differences in using the Internet, and gender differences in online education performance. Based on the review, we proposed hypotheses about the direction of the gender differences and the potential influence of country-level characteristics on gender differences in the enrollment in and completion of STEM MOOCs.

### Cross-national differences in STEM enrollment and performance

One strand of previous empirical studies suggests that gender-equal cultures are associated with higher levels of female representation in STEM choices and smaller gender differences in STEM performance. Van Langen and Dekkers found that females from countries that were more gender conscious and advanced in females’ emancipation (e.g., Sweden) considered STEM courses more attractive [17]. In terms of STEM performance, previous studies showed that there were smaller gender differences in math performance in more gender-equal cultures [18,19]. On the other hand, conservative social norms and cultural expectations may both decrease the likelihood that females will choose STEM courses and undermine their performance in STEM. Nosek and colleagues found that national-level implicit gender stereotypes are positively associated with a national-level male-favoring gender gap in 8^th^-grade science and math achievement [20]. McDaniel found that the male-favoring gender gap in STEM career expectation became larger in countries with more traditional gender ideologies [21]. If this pattern were the norm, we would expect the gender gap in STEM MOOC enrollment and completion to decrease as the level of gender equality in a country increases.

Another strand of empirical studies found that economic development is negatively associated with females’ participation in STEM field [22]. Bradley found that the proportion of females in engineering was higher in the less economically developed countries than in more economically developed countries [22]. For instance, Mexico had the highest percentage of tertiary computing degrees awarded to females in 2011 among countries that are members of the Organization for Economic Co-operation and Development (OECD) [23]. In addition among 44 countries, Finland was found to have the highest level of gender segregation in fields of study [24]. The pronounced gender segregation in economically developed countries may be accounted for by the varying opportunities to express a gendered identity and the cultural beliefs that males and females are fundamentally and innately different [24]. Females from developed countries may feel that it is legitimate to express their aversion to math or STEM-related courses, which reinforces their inclination to avoid STEM fields. If this were the case, we would expect more gender segregation in STEM MOOC enrollment and completion in more economically developed countries than in other countries.

When it comes to developing countries, lack of access to high-quality STEM courses has been one of the factors that has hindered students’ enrollment in traditional STEM fields [17]; this may be especially true for females from developing countries. In addition to local programs to promote STEM education in developing countries [25], the free and easy access to online courses provided by elite universities may spark the interest of learners in developing countries to pursue STEM education. Research shows that internet users, especially females from developing countries, were more interested in working in STEM fields than their peers in developed countries [26]. For instance, 77% of female respondents from developing countries stated that they felt encouraged to work in STEM fields while only 46% of female respondents from developed countries felt the same way [26]. Based on this, we may expect smaller gender differences in STEM MOOC enrollment and completion in less developed countries than developed countries.

### Gender differences in using the Internet

The male-favoring gender differences in the use of computers, mobile devices, and the Internet still exist in most parts of the world, especially in developing countries [27–31]. For instance in 2013, it was reported that the male-favoring gender gap was larger in developing countries, where 16% fewer females than males used the Internet compared with only 2% fewer females than males did so in developed countries [30]. In 2016, the regional gender gap was largest in Africa (23%) and smallest in the Americas (2%) [32]. Hilbert found that fewer females accessed and used Information and Communication Technology (ICT) than did males in developing countries [33]. Another report showed that in developing countries females were 50% less likely to access the Internet than were males in the same age group with similar levels of education and household income [34]. Based on this, we may expect that females are less likely than males to enroll in STEM MOOCs and that larger male-favoring gender differences in STEM MOOC enrollment exists in less developed countries than developed countries.

### Gender differences in online education performance

Previous studies show that females perform as well as, if not better than, males in online learning settings. For instance, Yukselturk and colleagues did not find significant differences in programming achievement with respect to gender in a self-regulated online learning environment in Turkey [35]. Wladis and colleagues found that females and males had similar success rates in online STEM courses provided by an urban community college in the United States [36]. Price reported that females studying online are confident and independent learners who may outperform their male counterparts in an online undergraduate course provided by Open University [37]. Chyung found that females scored higher than males in a graduate-level online course provided by a mid-sized university in the United States [38]. Xu and Jaggars found that females outperformed males in online courses provided by 34 community and technical colleges in Washington State [39]. Based on this, we may expect that once females enroll in STEM MOOCs, they may be equally or more likely than males to complete them.

In summary, previous studies suggest possible gender differences in STEM MOOC enrollment and completion as well as varying gender differences associated with country-level characteristics (e.g., gender equality and economic development level). We ask the following research questions: 1. What are the directions of gender differences in STEM MOOC enrollment and completion? 2. How do country-level characteristics (e.g., gender equality and economic development level) moderate the relationship between gender and the enrollment in and completion of STEM MOOCs? If MOOCs were to hold the promise to democratize and empower the traditionally disadvantaged females, the potential gains would be much larger in less gender-egalitarian and less economically developed countries.

## Materials and methods

To address our research questions, we used the HarvardX-MITx Person-Course de-identified dataset from the 2012–2013 academic year (Fall 2012, Spring 2013, Summer 2013) (MITx and HarvardX, 2014), which included 16 HarvardX and MITx courses on the edX platform. This dataset is the most comprehensive publicly available dataset on MOOCs. In total, 13 MOOCs were labeled as STEM MOOCs and three MOOCs were labeled as non-STEM. Table 1 presents the description of the courses in the dataset. Courses in Biology, Computer Science, Engineering and Mechanics, Mathematics and Statistics, Physics, Chemistry, and Environmental Studies were labeled as STEM MOOCs because these fields are included in the STEM Designated Degree Program List [40]. Learners in these online courses came from all over the world. The dataset included self-reported variables such as gender, age, highest level of education, country, and information about the courses that learners enrolled in and whether they have completed those courses. There were 641,138 person-course observations in the original dataset. We aggregated the dataset and obtained 476,532 unique students’ observations. After removing those who did not report specific country names such as "other Europe" and personal information such as age, gender, and highest level of education and those who reported age under 10, we obtained 269,263 student observations from 25 countries for data analysis. The dependent variable STEM MOOC enrollment was set to 1 if a learner took at least one STEM MOOC and 0 otherwise. The dependent variable STEM MOOC completion was set to 1 if a STEM MOOC enrollee completed least one STEM MOOC and 0 otherwise.

**Table 1:** Course description.

Institution	Course Title	STEM MOOC
HarvardX	The Ancient Greek Hero	No
HarvardX	Introduction to Computer Science I	Yes
HarvardX	Justice	No
HarvardX	Health in Numbers: Quantitative Methods in Clinical & Public Health Research	Yes
HarvardX	Human Health and Global Environmental Change	Yes
MITx	The Challenges of Global Poverty	No
MITx	Elements of Structure	Yes
MITx	Introduction to Solid State Chemistry 01	Yes
MITx	Introduction to Solid State Chemistry 02	Yes
MITx	Circuits and Electronics 01	Yes
MITx	Circuits and Electronics 02	Yes
MITx	Introduction to Computer Science and Programming 01	Yes
MITx	Introduction to Computer Science and Programming 02	Yes
MITx	Introduction to Biology–The Secret of Life	Yes
MITx	Electricity and Magnetism	Yes
MITx	Mechanics Review	Yes

We used the Gender Gap Index (GGI) created by the World Economic Forum to measure a country’s gender equality level [41]. GGI reflects the gap between males and females in access to resources and opportunities for health, educational attainment, economic participation, and political empowerment, and was used as a key predictor variable in our models. The GGI is composed of the country’s health index, educational attainment index, economic participation index, and political empowerment index. The health index refers to the sex ratio at birth and the gap between females’ and males’ healthy life expectancies. The educational attainment index reflects the ratios of females to males in primary-, secondary-, and tertiary-level education. The economic participation index reflects the gap between females’ and males’ labor force participation rates, wage equality, and the ratio of females to males among professional workers and senior officials. The political empowerment index reflects the gender gap at the highest-level of political decision-making [41]. GGI ranges from 0 (full inequality) to 1 (full equality) with a higher GGI referring to a more gender-egalitarian environment. The GGI for the 25 countries in the dataset ranges from 0.55 (Pakistan) to 0.78 (Philippines). For this study, we used the grand mean centered GGI as a level 2 variable in the multilevel models.

As GGI does not reflect a country’s development level, we included GDP per capita (2012) to measure a country’s economic development level the year the data were collected [42] and used the grand mean centered log GDP per capita as a level 2 variable in the analysis. The GDP per capita for the countries in the dataset ranged from $859 (Bangladesh) to $67,512 (Australia). We also included controls for the learner’s age and education using a bachelor’s degree as the reference group.

To answer our research questions on the directions of gender differences and how country-level characteristics (e.g., gender-equal culture and economic development level) may moderate the relationship between gender and the enrollment in and completion of STEM MOOCs, we conducted a series of multilevel logistic regression models using R lme4 package to account for the nesting of an individual within a country. The multilevel framework is an appropriate method for addressing our research questions because it takes into account the nesting of individuals within groups (in our case within countries) [43]. These models allow for the examination of how country-level variables (e.g., GGI) are associated with individual’s enrollment in and completion of STEM MOOCs as well as with cross-level interaction effects between individuals and country-level variables. The model examining GGI can be written:

Level-1 equation

$${Outcome}_{ik}=\beta_{0k}+\beta_{1k}{Female}_{ik}+\beta_{2k}{Age}_{ik}+\beta_{3k}{Education}_{ik}+e_{ik}$$

Level-2 equation

$$\beta_{0k}=\gamma_{00}+\gamma_{01}{GGI}_{k}+u_{0k}$$

$$\beta_{1k}=\gamma_{01}+u_{1k}$$

The level 1 equation indicates that the learner’s outcome is a linear combination of the intercept for the country where the learner comes from (*β*_0*k*_), the main effect of being female (*β*_1*k*_*Female*_*ik*_), the main effect of age (*β*_2*k*_*Age*_*ik*_), the main effect of education (*β*_3*k*_*Education*_*ik*_), and a residual for the learner (*e*_*ik*_). The level-two equation allows for random variations in intercepts between countries where the country-level intercepts (*β*_0*k*_) are comprised of a grand mean (*γ*_00_), a fixed effect for GGI (*γ*_01_*GGI*_*k*_), and random deviations in intercepts between countries (*u*_0*k*_). Additionally, a random effect for gender was included such that the association between gender and the outcome was allowed to differ between countries as denoted by *u*_1*k*_. For the models examining the relation between GDP per capita and STEM MOOC enrollment and completion, GDP per capita instead of GGI is used in the above-mentioned equations.

We tested all of our models for the inclusion of random slopes and random intercepts. Using the likelihood ratio test, we found that random slope models performed significantly better when a random slope was included for female. Therefore we report results from models where slopes were able to vary randomly for female. To examine the degree to which learners from different countries differ in their propensity to choose and complete STEM MOOCs, we calculated the intraclass correlation coefficient (ICC) to determine if there was sufficient country-level variance to model [44]. The ICC is 0.2 for enrollment and 0.13 for completion, indicating that about 20% and 13% of the variation in STEM MOOC enrollment and completion, respectively, can be attributed to differences in learners’ country of origin. We first ran multilevel logistic regression models for STEM MOOC enrollment, and then examined only those learners who took at least one STEM MOOC and modeled their STEM MOOC completion.

## Results

### Descriptive analysis

**Fig 1:**
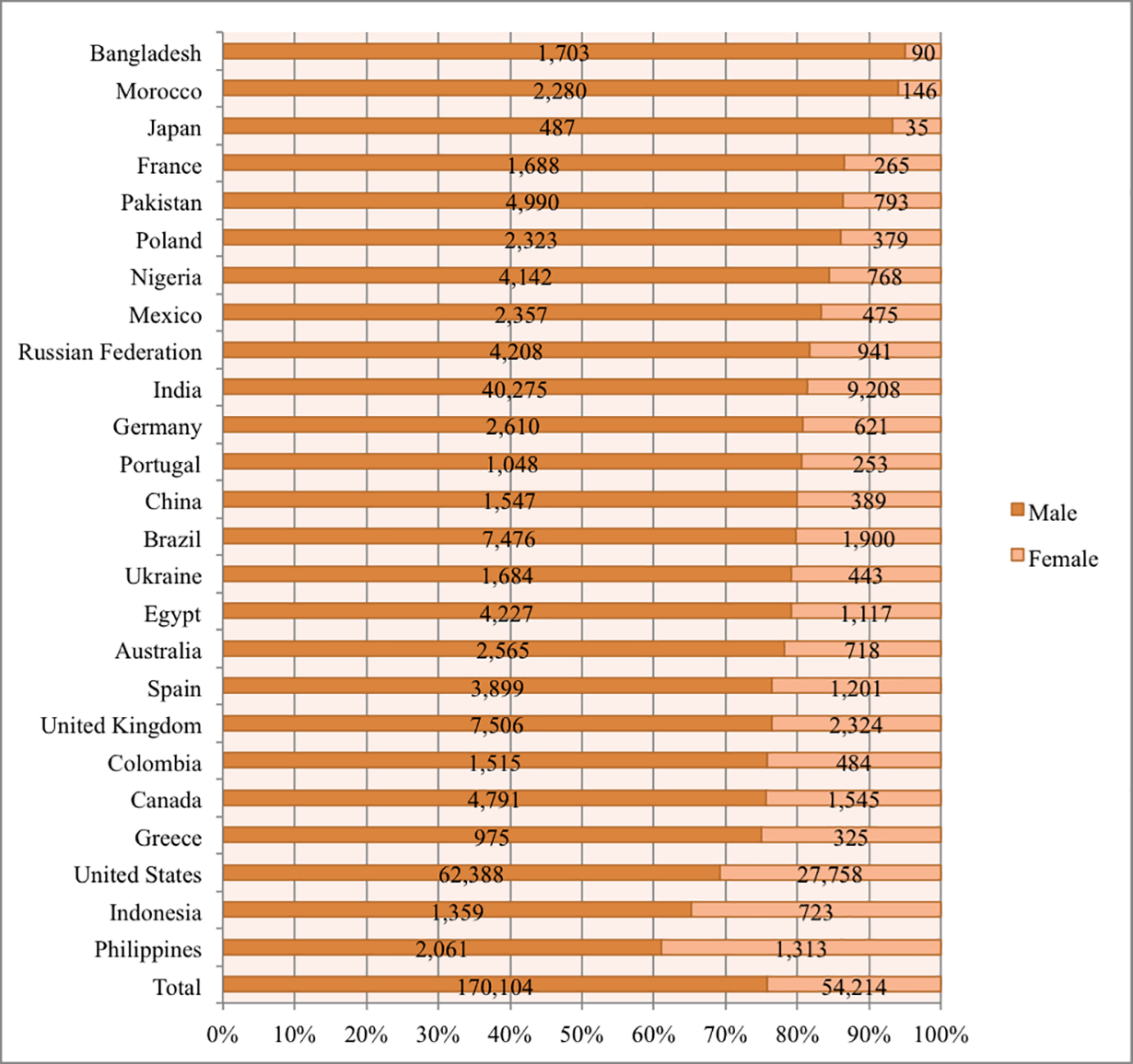
Number of males and females who enrolled in one or more STEM MOOCs in each country.

**Fig 2:**
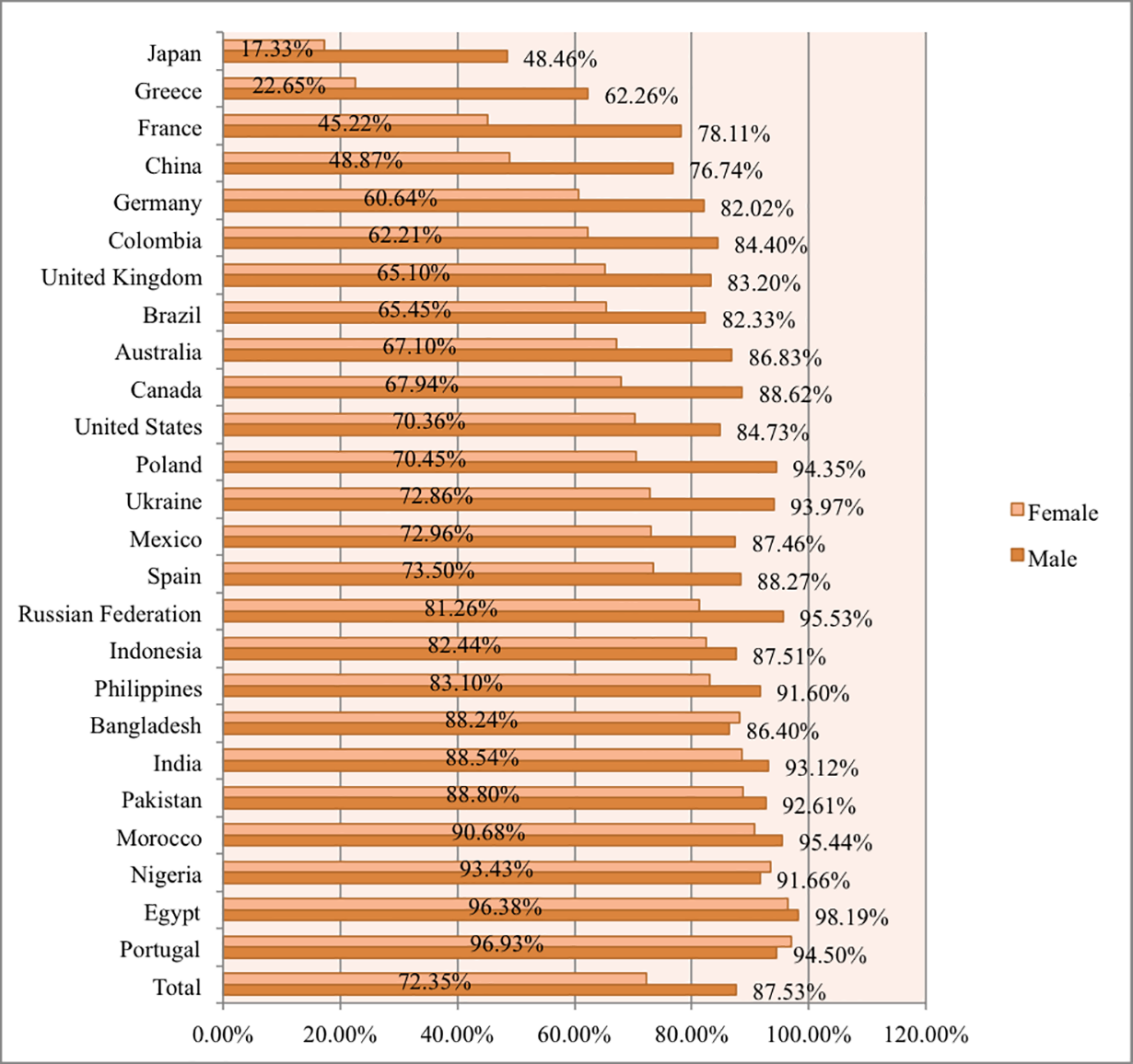
Percentage of all MOOC enrollees in each country who enrolled in one or more STEM MOOCs, by gender.

Fig 1 displays the number of female and male learners who took at least one STEM MOOC in each country. Across all the countries, 54,214 female learners chose to enroll in at least one STEM MOOC, which comprised of 24.16% STEM MOOC learners (n = 224,318) in the dataset. By country, the percentage of STEM MOOC learners who were female ranged from 5% in Bangladesh to 38.92% in the Philippines. It is worth noting that the top two countries with the highest female representation were developing countries (the Philippines and Indonesia). Fig 2 shows the percentage of all MOOC learners in each country who enrolled in at least one STEM MOOC, by gender. Across all the countries, 72.35% of female and 87.53% of male MOOC learners enrolled in at least one STEM MOOC. The percentage of female MOOC learners taking one or more STEM MOOCs ranged from 17.33% in Japan to 96.93% in Portugal. In several countries (including Portugal, Egypt, and Nigeria), female learners took STEM MOOCs at nearly the same rate as males. For example, 96.38% of female and 98.19% of male MOOC learners from Egypt chose to enroll in at least one STEM MOOC. This shows that while a lower percentage of female MOOC students overall enrolled in STEM MOOCs, the gender differences varied considerably by country.

**Fig 3:**
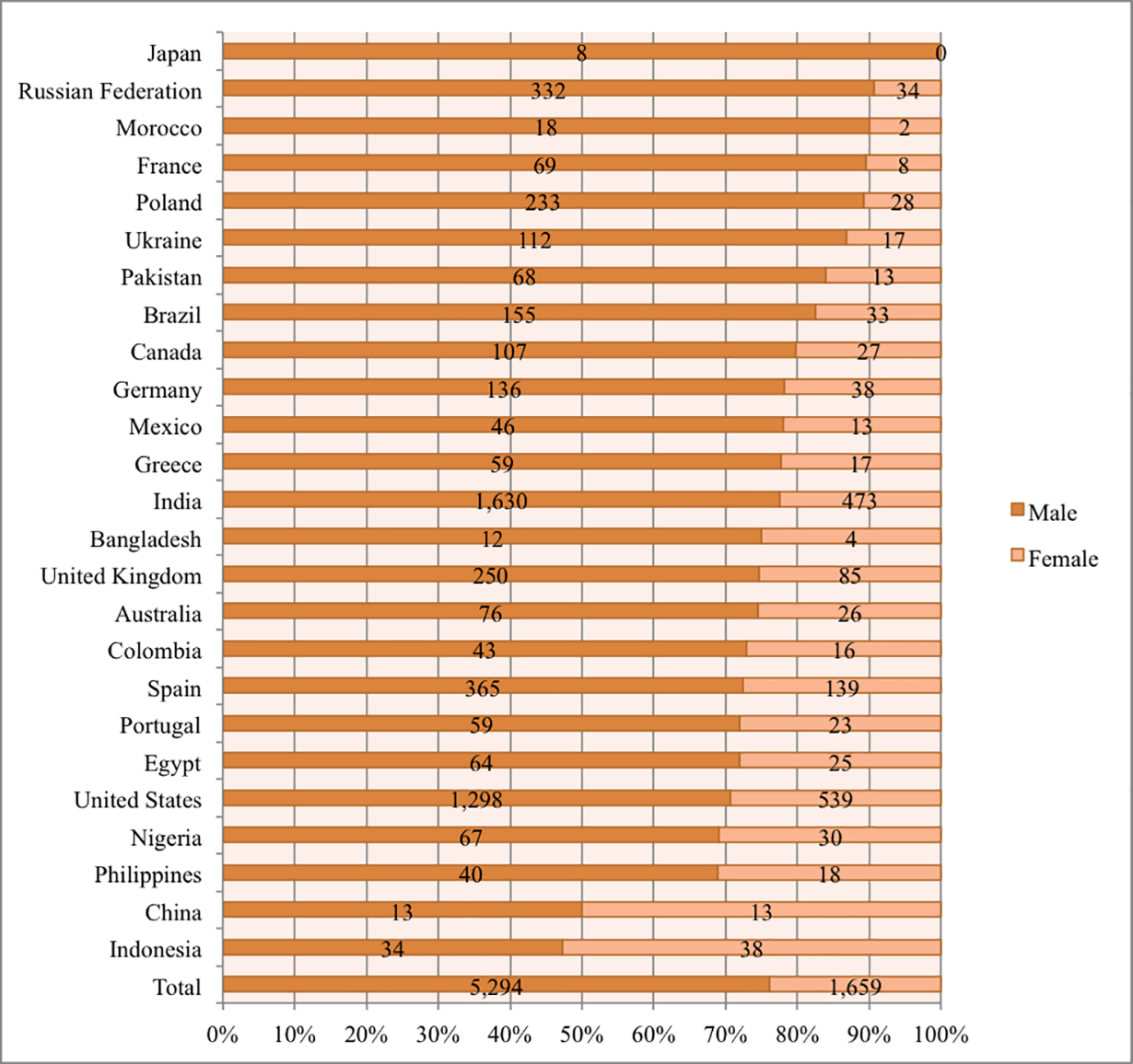
Number of males and females who completed one or more STEM MOOCs in each country.

**Fig 4:**
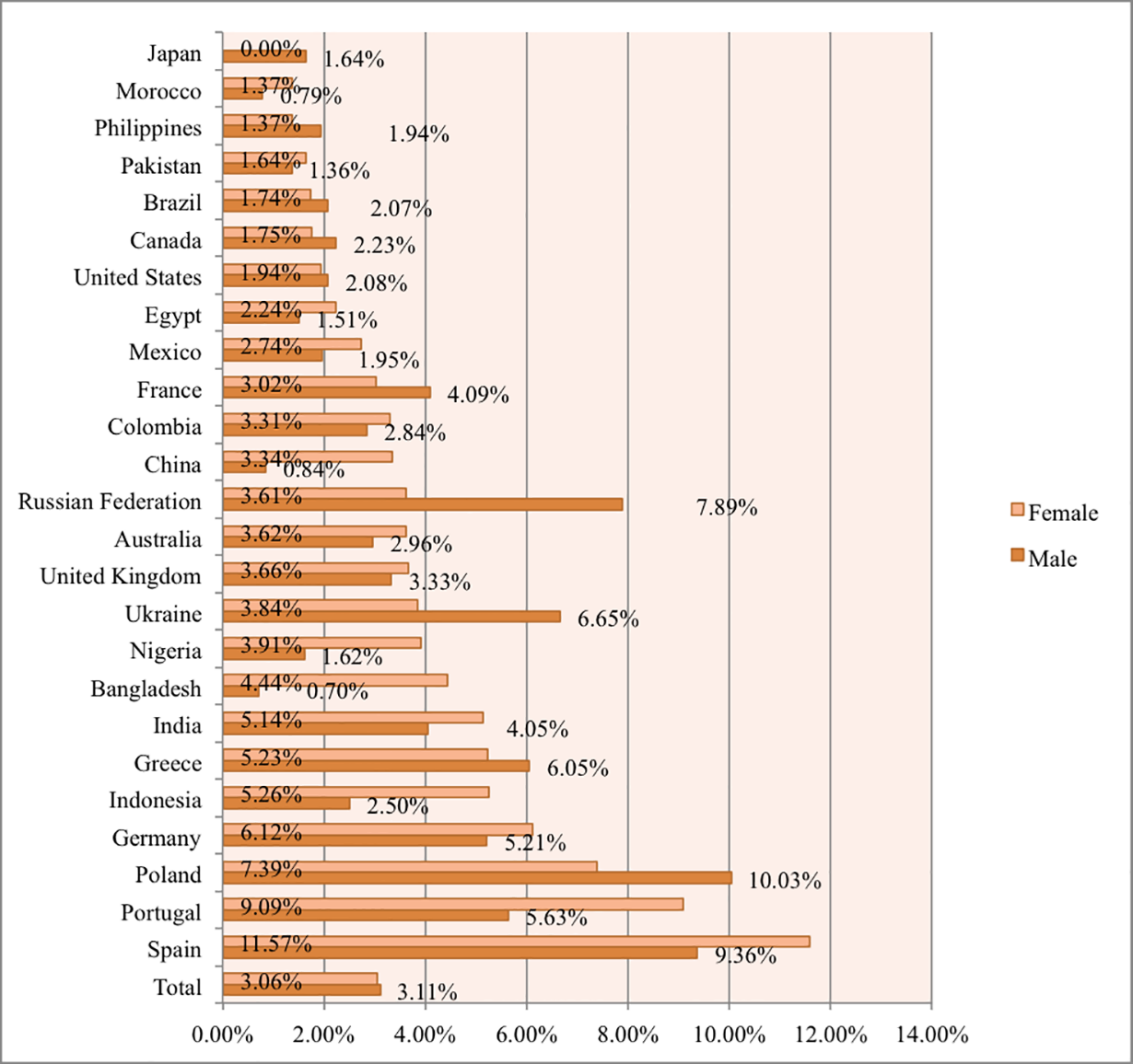
Percentage of STEM MOOC enrollees in each country who completed one or more STEM MOOC, by gender.

When it comes to completion of STEM MOOCs (Fig 3), only 1,659 female and 5,294 male STEM MOOC learners completed at least one STEM MOOC. On average, 23.86% of STEM MOOC learners who completed a MOOC were female, but this varied greatly by country. As shown in Fig 3, Indonesia, China, and the Philippines had the highest rate of females completing at least one STEM MOOC, with 52.78%, 50%, and 31.03%, respectively. When examining the STEM MOOC completion rate by gender alone (see Fig 4 Total), only 3.06% of females and 3.11% of males who enrolled in STEM MOOCs actually completed at least one STEM MOOC. This suggests that both males and females had low completion rate in STEM MOOCs while these rates varied across countries.

### Statistical analysis

Table 2 shows the results from the multilevel logistic regression models used to assess the relationship between gender and STEM MOOC enrollment, and the moderation effect of country-level characteristics (e.g., GGI and GDP per capita). Seven models are presented each with an increasing number of covariates (the same models were conducted for STEM MOOC completion, see Table 3). Model 1 tested the raw effect of being female on the enrollment in STEM MOOCs. Model 2 controlled for age and Model 3 controlled for both age and the education level. Based on Model 3, Model 4 controlled for GGI and Model 5 included the interaction term between female and GGI. Model 6 controlled for log GDP per capita and Model 7 controlled for the interaction term between female and log GDP per capita. Across the entire sample, a female’s probability of enrolling in at least one STEM MOOC was 12% lower than that of a male, when controlling for the individual’s age and highest level of education, as shown by Model 3 in Table 2. Model 3 in Table 2 also shows that age was negatively related to enrollment in STEM MOOCs (r = -0.003, p < 0.001). Learners with a less than secondary degree (r = -0.01, p < 0.1), a master’s (r = -0.03, p < 0.001), or a Ph.D degree (r = -0.01, p < 0.001) were less likely than those with only a bachelor’s degree to enroll in STEM MOOCs while learners with a secondary education (r = 0.03, p < 0.001) were more likely than those with a bachelor’s degree to enroll in STEM MOOCs (see Model 3 in Table 2). GGI (r = -0.51, p < 0.001) was negatively significantly associated with enrollment in STEM MOOCs (see Model 4 and Model 5 in Table 2). The negative interaction term between female and GGI (r = -0.42, p < 0.001) was significant, which indicates that higher gender equality was related to an increased gender gap in STEM MOOC enrollment (Model 5 in Table 2). More specifically, a 0.1 increase of GGI is associated with a 5.1% decrease in an enrollee’s probability and an additional 4.2% decrease in a female’s probability to enroll in STEM MOOCs. GDP per capita was negatively associated with STEM MOOC enrollment (r = -0.03, p < 0.001) (see Model 7 in Table 2). In addition, higher GDP per capita is associated with an increased gender gap in STEM MOOC enrollment when controlling for age and the highest level of education, as shown by Model 7 in Table 2. More specifically, a 1% increase in GDP per capita is associated with a 3% decrease in an enrollee’s probability and an additional 3% decrease in a female enrollee’s probability of STEM MOOC enrollment. The findings suggest that the male-favoring gender differences in STEM MOOC enrollment were smaller in less gender-equal and less economically developed countries.

**Table 2:** Multilevel logistic regression on whole sample for enrolling in STEM MOOCs.

	Model 1	Model 2	Model 3	Model 4	Model 5	Model 6	Model 7
Female	-0.13***	-0.13***	-0.12***	-0.12***	-0.12***	-0.12***	-0.12***
	(0.02)	(0.02)	(0.02)	(0.02)	(0.02)	(0.02)	(0.00)
Age		-0.004***	-0.003***	-0.003***	-0.003***	-0.003***	-0.003***
		(0.00)	(0.00)	(0.00)	(0.00)	(0.00)	(0.00)
< Secondary			-0.01^+^	-0.01^+^	-0.01^+^	-0.01^+^	-0.01^+^
			(0.01)	(0.01)	(0.01)	(0.01)	(0.01)
Secondary			0.03***	0.03***	0.03***	0.03***	0.03***
			(0.00)	(0.00)	(0.00)	(0.00)	(0.00)
Master			-0.03***	-0.03***	-0.03***	-0.03***	-0.03***
			(0.00)	(0.00)	(0.00)	(0.00)	(0.00)
PhD			-0.01***	-0.01***	-0.01***	-0.01**	-0.01**
			(0.00)	(0.00)	(0.00)	(0.00)	(0.00)
GGI				-0.38^+^	-0.51***		
				(0.20)	(0.08)		
Female*GGI					-0.42***		
					(0.06)		
Log GDP per capita						-0.03	-0.03*
						(0.02)	(0.02)
Female*log GDP per capita							-0.03*
							(0.01)
N	269,263	269,263	269,263	269,263	269,263	269,263	269,263
Marginal R^2^	0.04	0.06	0.06	0.08	0.10	0.09	0.12
Conditional R^2^	0.24	0.26	0.26	0.26	0.27	0.25	0.27

*Note*. Standard errors in parentheses.

Coefficients are average marginal effects.

The R^2^ given above is Nakagawa and Schielzeth’s R^2^ [45].

***p < 0.001.

**p < 0.01.

*p < 0.05.

^+^p < 0.1.

**Table 3:** Multilevel logistic regression for completing conditional on STEM MOOC enrollment.

	Model 1	Model 2	Model 3	Model 4	Model 5	Model 6	Model 7
Female	0.002	0.002	0.002	0.002	0.001	0.002	0.001
	(0.00)	(0.00)	(0.00)	(0.00)	(0.00)	(0.00)	(0.00)
Age		-0.00^+^	-0.00***	-0.00***	-0.00***	-0.00***	-0.00***
		(0.00)	(0.00)	(0.00)	(0.00)	(0.00)	(0.00)
< Secondary			0.01*	0.01*	0.01*	0.01*	0.01*
			(0.00)	(0.00)	(0.00)	(0.00)	(0.00)
Secondary			0.00	0.00	0.00	0.00	0.00
			(0.00)	(0.00)	(0.00)	(0.00)	(0.00)
Master			0.01***	0.01***	0.01***	0.01***	0.01***
			(0.00)	(0.00)	(0.00)	(0.00)	(0.00)
PhD			0.03***	0.03***	0.03***	0.03***	0.03***
			(0.00)	(0.00)	(0.00)	(0.00)	(0.00)
GGI				0.11***	0.17**		
				(0.03)	(0.06)		
Female*GGI					-0.11***		
					(0.03)		
Log GDP per capita						0.01^+^	0.01*
						(0.00)	(0.00)
Female*log GDP per capita							-0.005^+^
							(0.00)
N	224,318	224,318	224,318	224,318	224,318	224,318	224,318
Marginal R^2^	0.001	0.001	0.01	0.02	0.03	0.02	0.03
Conditional R^2^	0.13	0.13	0.13	0.12	0.13	0.12	0.12

*Note*. Standard errors in parentheses. Coefficients are average marginal effects. The R2 given above is Nakagawa and Schielzeth’s R2 [45].

***p < 0.001.

**p < 0.01.

*p < 0.05.

+p < 0.1.

Table 3 shows the results of using multilevel logistic regression models to assess the relationship between being female and STEM MOOC completion, and the moderation effect of country-level characteristics (e.g., GGI and GDP per capita). We found that females and males are equally likely to complete, after controlling for age, highest level of education, country-level characteristics, and the interaction term between female and country-level variables (see Model 1–7 in Table 3). Furthermore, increased gender equality (GGI) (r = 0.17, p < 0.01) was positively associated with the completion of STEM MOOCs, i.e., 0.1 increase of GGI increase the probability to complete STEM MOOCs by 1.7%. The interaction term between gender and GGI (r = -0.11, p < 0.001) was negatively associated with completion of STEM MOOCs, indicating that a 0.1 increase of GGI is associated with a 1.1% decrease in a female’s probability to complete STEM MOOCs (see Model 5 in Table 3). GDP per capita was positively associated with learners’ completion of STEM MOOCs (r = 0.01, p < 0.5) and reduced female advantage in completing STEM MOOCs (r = -0.005, p < 0.1) (see Model 7 in Table 3). Precisely, a 1% increase in GDP per capita increases the probability to complete STEM MOOCs by 1% and decreases female’s probability to complete STEM MOOCs by 0.5%, as shown by Model 7 in Table 3. The findings suggest that the gender difference in STEM MOOC completion is smaller in less gender-egalitarian and economically developed countries.

## Discussion and conclusions

This study complements previous work investigating the democratization of MOOCs in the United States [5], by suggesting that MOOCs have the potential to democratize education across the world and provide STEM learning opportunities for learners, particularly female learners from less gender-equal and less economically developed countries. This study demonstrates that while females were less likely than males to enroll in STEM MOOCs, females and males were equally likely to complete them. A higher probability to enroll in STEM MOOCs and smaller male-favoring gender gaps in STEM MOOC enrollment and completion were found in less gender-egalitarian and less economically developed countries.

Considering that females are generally less likely than males to enroll in STEM MOOCs and only consisted of 24% of STEM MOOC learners, more studies should be conducted to explore the factors influencing females’ enrollment in STEM MOOCs. Currently, it is unclear whether females’ underrepresentation in STEM MOOC enrollment is due to the lack of access to the Internet [30], gender stereotypes related to STEM field [24], not being aware of online STEM learning opportunities, or other factors. Knowing the underlying cause of female underrepresentation in enrollment would allow for targeted corrective action. Corresponding actions can be taken to increase females’ enrollment in STEM MOOCs based on the underlying reasons. For instance, if females’ low participation is due to the fact that they are not aware of the opportunities of taking free online STEM courses or the opportunities and financial rewards that could result from taking these courses [12,46], additional outreach could promote such awareness.

The smaller male-favoring gender gaps in STEM MOOC enrollment and completion in less gender-egalitarian and less economically developed countries indicate that MOOCs might offer broad country-level social benefits for less socially and economically developed countries. Free and easy access to MOOCs in developing countries allows females to try out STEM courses that are not easily available to them in their local communities. This finding also aligns with the educational-gender-equality paradox found by Stoet and Geary, i.e., the gender differences in the magnitude of relative academic strengths and pursuit of STEM degrees rose with increases in national gender equality [47].

These phenomena can be explained by the expectancy value theory [11]. The life-quality pressures in less gender-equal and less economically developed countries may increase females’ utility value of pursuing a STEM education and career, which in turn promotes females’ STEM engagement [48]. Pursuing a STEM education and career may be more appealing to females from less socially and economically developed countries, because STEM occupations are usually well paid and can provide economic security. On the other hand, the cost for females from more socially and economically developed countries to forgo a STEM career is relatively small, since there may be a higher level of social and economic security [47]. At the same time, females from more developed countries may be more influenced by gender essentialist ideology [22,24], which in turn reduces their interest and engagement in STEM. We suggest that future studies be conducted to understand females’ decision-making process to enroll in and complete STEM MOOCs.

This study has certain limitations. First, the fact that the pseudo-R squareds are small (see Tables 2 and 3) implies that the variables in the model only explain a portion of the overall variance in STEM MOOC enrollment and completion. Though this is a limitation, the paper focuses on the narrower question of the moderating effect of country-level characteristics on the relationship between gender and enrollment or completion of STEM MOOCs. In that sense, the pseudo-R squareds, though small, are still scientifically valid for identifying the moderator.

Secondly, the datasets were collected in 2012–2013 and thus do not reflect more recent trends in MOOC enrollment and completion. This is due to the nature of MOOC data that has been made publicly available so far. As additional MOOC data becomes available, future research should investigate whether and how the patterns of results identified in our study might change.
